# Characteristics of Helicopter Emergency Medical Services (HEMS) Interventions for Burn Patients—A Four-Year Retrospective Analysis

**DOI:** 10.3390/jcm13247738

**Published:** 2024-12-18

**Authors:** Arkadiusz Wejnarski, Piotr Konrad Leszczyński, Maja Biadun, Maria Malm, Kryspin Mitura, Daryna Sholokhova, Patryk Rzońca, Robert Gałązkowski, Leszek Marzec

**Affiliations:** 1 Department of Medical and Health Sciences, University of Siedlce, 08-110 Siedlce, Poland; arkadiusz.wejnarski@uws.edu.pl (A.W.);; 2 Department of Medical Informatics and Statistics with e-Health Lab, Medical University of Lublin, 20-090 Lublin, Poland; maria.malm@umlub.pl; 3 Department of Human Anatomy, Faculty of Health Sciences, Medical University of Warsaw, 02-091 Warsaw, Poland; 4 Department of Medical and Health Sciences Tarnobrzeg, State Higher Vocational School Memorial of Prof. Stanislaw Tarnowski in Tarnobrzeg, 39-400 Tarnobrzeg, Poland

**Keywords:** burns, trauma, emergency medical service (EMS), helicopter emergency medical service (HEMS), treatment, physician

## Abstract

**Background:** The World Health Organization (WHO) estimates that 180,000 patients die from burns every year, which is considered a serious public health issue. Patients with burns require immediate pre-hospital care and transport to specialized treatment facilities. The aim of this study was to outline the profile of the burn patient from the perspective of the Polish Medical Air Rescue (PMAR), as well as to analyze the medical procedures being implemented. **Methods:** The study includes 2154 interventions by air emergency medical teams (AEMS) which provided aid for burn patients. The analysis covered the period from 2018 to 2022, including nationwide data made available from the IT systems of the PMAR. Statistical design was used, allowing for correlations of variables, at a significance level of *p* < 0.05. **Results:** Patients’ ages ranged from 1 month to 96 years (mean 35.05; SD ± 26.88). Adult patients (*n* = 1409; 65.41%) constituted the vast majority. The number of interventions to children below 1-year-old was noticeable (*n* = 394; 18.29%). Men were the most likely to suffer burns, up to three times more often than women (*n* = 1574, 73.07% vs. *n* = 570, 26.46%. T29—burns to multiple body areas (*n* = 890)—and T21—burns to the trunk (*n* = 255)—were most frequently reported as diagnoses according to the ICD-10 classification. A statistically significant association was found between age group and ICD-10 diagnosis (*p* < 0.001). The vast majority of patients were transported from the scene directly to Burn Treatment Centers (*n* = 1373; 63.74%). Treatment of pain by helicopter emergency medical services (HEMS) crews appeared to be effective (*p* < 0.001), and other interventions consisted of administering medications—ketamine (23.72%), rocuronium bromide (15.78%), propofol (14.02%)—and procedures such as sedation (30.87%), as well as intubation (13.42%) and mechanical ventilation (13.23%). **Conclusions:** The burn patient profile indicates men with a mean age of 35 years. Nevertheless, HEMS crews often carry out missions to infants and newborns. The most common diagnosis was extensive body burns. In 63.74% of the missions, patients were transported to the Burn Treatment Center. The HEMS crews implement effective pharmacological analgesia, and handle rescue medications and procedures to stabilize the patient’s condition.

## 1. Introduction

Tissue injuries caused by factors such as temperature, chemicals, radiation, friction, and electricity cause burns [1]. The World Health Organization (WHO) estimates that 180,000 patients die from burns every year, which remains a major public health burden [2]. This phenomenon mainly affects low- and middle-income countries, but globally, non-fatal burns are a common cause of morbidity, long-term hospitalization, body disfigurement, and disability. However, the aesthetic-related aspects cause stigma and psychological problems in burn patients. In developed countries, there has been a tendency toward treating patients in specialized medical center settings, allowing for shorter hospital stay as well as improved outcomes [3].

Classifying burns based on their extent and depth is a key part of diagnostics and treatment planning. The commonly used Abbreviated Burn Severity Index (ABSI) procedure is a five-variable scale to help assess burn severity. The variables considered are sex, age, presence of inhalation injury, presence of a full-thickness burn, and percentage of total body surface area burned. Third-degree burns (depth—deep dermal; color—blotchy red) and, sometimes distinguished, fourth-degree burns (depth—full thickness; color—white) are the most serious injuries, involving destruction of the dermis along with deep tissue. This is the condition wherein necrotic and sometimes charred areas are formed. Hospitalization is essential in this case, and treatment often requires skin and tissue replantation to restore lost structures. The treatment process is extremely long and expensive. Optimal burn management requires a comprehensive clinical analysis that not only includes the extent of tissue damage, but also the patient’s characteristics and disease burden. Individualization of care is key, and the age of the patient affects the time of recovery [4]. The procedure of HEMS teams in Poland is described in the internal procedure for burn patients and was prepared according to the recommendations of the American Burn Association (ABA).

All scientific opinions agree that burn-related complications can be minimized by proper and prompt first aid [5]. In addition to the actions taken by witnesses to the incident (including interrupting the process causing the burn, cooling with water), further medical management both in the pre-hospital settings and the performance of specialized procedures and treatment in medical facilities is crucial. The primary role is played by the time between the incident and the initiation of therapy. With that in mind, there are protocols developed for emergency services to conduct an initial examination, secure injuries, provide pain management, and allocate the patient to a dedicated hospital [6]. These tasks are usually performed by emergency medical services, which in Europe most often operate as ground-based emergency medical services (EMS) [7] and air emergency medical services (AEMS) [8]. In Poland, AEMS carry out HEMS missions [9]. AEMS crews consist of a professional medical team (medical doctor and paramedic/nurse), as well as a pilot [10]. The specifics of ground teams, which most often do not include a doctor, are different [11,12,13].

Currently, there are 22 AEMS bases in Poland (including one seasonal base) ready to carry out HEMS missions with EC135 helicopters. The HEMS team crew consists of a pilot, a paramedic/nurse, and a physician. In addition, AEMS has two jet aircraft for long-distance transport and Robinson R-44 helicopters. HEMS missions are ordered by medical dispatchers. Decisions on transporting a patient to a dedicated medical facility are made by the crew physician. There are 11 burn treatment centers in Poland, the list of which and the current number of vacancies are posted on the website www.lpr.com.pl (accessed on 1 December 2024). Additionally, some hospitals perform shifts and have a burn unit in their resources.

The authors attempted in this study to outline the profile of a burn patient from an HEMS perspective, as well as to identify the medical procedures implemented and the transport to hospital. Both the medical activities carried out at the scene by the medical team and the target transport site were analyzed, including the Burn Centers (BC).

## 2. Materials and Methods

The study material consisted of medical and operational records of the AEMS of 1 January 2018 to 31 December 2022. The scope included all missions performed in the country for burn patients. The approval of the unit’s administrator, as well as the ethics committee No. 4/2024 of the University of Siedlce, were obtained. The data were anonymized and processed digitally. The inclusion criteria were missions containing the term “burn” in the AEMS digital database—both in the documentation description and the diagnosis according to the ICD-10 classification. Cases in which the analyzed criteria were not described in the medical documentation were not included.

Statistical processing—results, relationships, and correlations were developed using spreadsheet Microsoft Excel 365 and Statistica 13.3. Sociodemographic data on patients, technical data on HEMS flights, and medical procedures implemented were compiled using counts and percentages for qualitative variables and descriptive statistics (mean, standard deviation, median, mode, minimum and maximum). Pearson’s Chi-square test was used to test the relationship between the two qualitative variables. Fisher’s exact test was chosen interchangeably when the expected counts were less than 5. Welch’s test was used to compare two groups and more than two groups—in several cases the distribution of the dependent variable was significantly different from normal, this was checked by the W Shapiro–Wilk test, but with the large groups (*n* > 30), a parametric test was chosen. The variances in the compared groups were heterogeneous. On the other hand, the Wilcoxon test was used to compare two dependent samples—the dependent variable in this case was ordinal. The significance level was set at *p* = 0.05.

## 3. Results

### 3.1. Characteristics of the Study Group

The total number of missions included in the analysis was 2154, selected from a digital database run by the Polish Medical Air Rescue. Patient ages ranged from 1 month to 96 years (mean 35.05; SD ± 26.88). Adult patients (*n* = 1409; 65.41%) constituted the vast majority. The number of interventions to children below 1-year-old—as many as 394 cases, or nearly one-fifth of all missions (18.29%)—is noticeable. Men suffered burns as much as three times more often than women (*n* = 1574, 73.07% vs. *n* = 570, 26.46%), as shown in Table 1.

Statistical analysis showed significant differences in age between patients according to ICD10 diagnoses (*p* < 0.001). It turned out that patients with the lowest age had a diagnosis of T21—thermal and chemical burn of the torso (Me = 1 year). In contrast, the oldest victims experienced thermal and chemical burns of the respiratory tract (Me = 51 years), as well as thermal burns classified by the extent of the body surface covered (Me = 45 years). Tukey’s post hoc test confirmed that age was significantly higher for T27 diagnoses and significantly lower for T21 than for most other diagnoses.

A statistically significant association was found between age group and ICD-10 diagnosis (*p* < 0.001). Pediatric patients were significantly more likely to experience thermal and chemical burns of the torso, i.e., T21 (27.19%), than adult patients (5.11%). In contrast, adults were far more likely to be diagnosed with T27: Thermal and chemical burns of the respiratory tract (5.11%), as well as T29—thermal and chemical burns to numerous areas of the body (47.13%). The results of the analysis are shown in Table 2.

### 3.2. Transport

The on-scene management of the air emergency medical team included not only transport to the treatment facility (*n* = 1679; 77.95%), but also the possibility of leaving the patient at the scene (*n* = 48; 2.23%), handing over to another AEMS team (*n* = 53; 2.46%), Polish Medical Air Rescue medical airplane (*n* = 36; 1.57%), and ground teams. This is related to ensuring the patient’s continuity of professional medical care in the pre-hospital setting, as well as the allocation of sometimes long distances for transfer to a higher referral center (Table 3).

The vast majority of patients were transported from the scene directly to Burn Treatment Centers (*n* = 1373; 63.74%), and slightly less than a third to another treatment facility (30.22%). An analysis by age was also performed, and it was found that as many as 77.86% of adult patients ended up in a Burn Treatment Center, while this percentage was significantly lower among pediatric patients, at 33.14% (Chi^2^ *p* < 0.001) (Table 4).

### 3.3. Pharmacological Treatment

**Figure 1:**
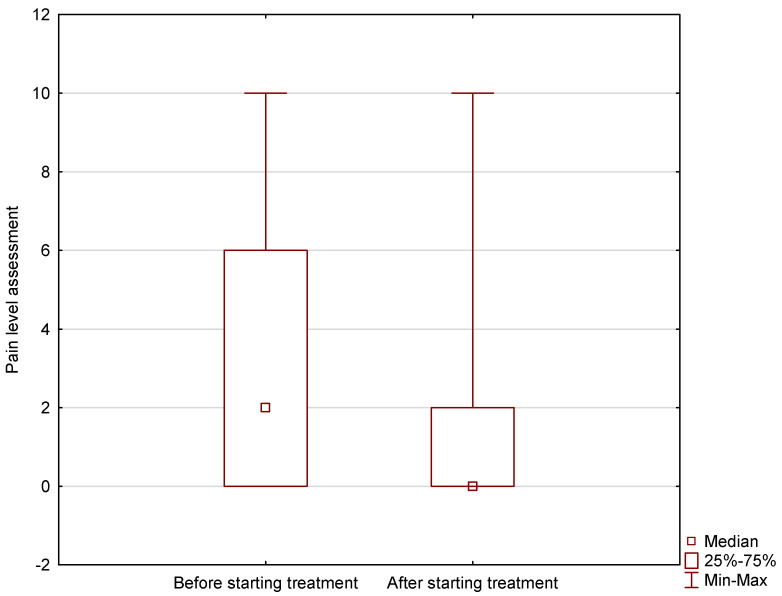
Pain level of burn patients before and after treatment implementation in AEMS settings.

Analysis of the NRS pain rating scale showed that the pain level of burn patients decreased significantly as a result of the treatment implementation by the Polish Medical Air Rescue (*p* < 0.001). Before treatment, the median pain score among patients was 2, and after treatment introduction, it was 0 (Figure 1).

In addition to anesthetic drugs, a number of other preparations were used in 43.13% of burn patients; the most common of which were ketamine (23.72%), rocuronium bromide (15.78%), and propofol (14.02%). A summary of the medications that the AEMS crew was authorized to administer is provided in Table 5.

### 3.4. Emergency Procedures

The list of procedures for which a medical doctor is authorized in Poland was selected to avoid discrepancies in the competence of representatives of other medical professions operating in the pre-hospital emergency system. The authors included missions that HEMS crews performed independently or by taking over a patient from a ground medical rescue team, who may have implemented some rescue procedures beforehand. Slightly less than half of burn patients (43.55%) required specialized medical treatments implemented by the HEMS team. The AEMS physician most often made the decision to perform the following: sedation (30.87%), as well as intubation (13.42%), mechanical ventilation (13.23%), and relaxation (8.40%). A detailed summary is provided in Table 6. In 22 cases, sudden cardiac arrest (SCA) was diagnosed, and resuscitation was implemented. In 7 patients (31.82%) with SCA, a mechanical chest compression device was used in the HEMS setting.

In a detailed analysis, the authors noted some correlations (Table 7). Adult patients were intubated almost seven times more often (18.15%) than pediatric patients (2.71%). The differences shown were statistically significant (*p* < 0.001). Statistical analysis showed a significant association between sex and intubation (*p* = 0.013). Men were intubated significantly more often than women (10.99% vs. 5.34%). The observed differences were found to be statistically significant (*p* < 0.001). RTS (*p* < 0.001) and GCS (*p* < 0.001) scores were significantly lower in burn patients who were intubated. Furthermore, intubation was used significantly more often in subjects with abnormal ECG (*p* < 0.001), dyspnea (*p* < 0.001), cyanosis (*p* = 0.001), and pale skin (*p* < 0.001).

## 4. Discussion

Individuals with body injuries constitute a significant group of patients requiring the aid of emergency medical teams [14]. Burns, due to their nature, are among the injuries that often generate the need to implement specialized treatments as early as possible in the pre-hospital settings, and then organize transfer to dedicated treatment centers. The authors analyzed a number of variables obtained from several years of medical records of a nationwide emergency unit in Poland—the Polish Medical Air Rescue [15].

The profile of the burn patient from the AEMS perspective indicated male sex (*n* = 1574; 73.07%) and a mean age of 35.05 years (SD ± 26.88). The study by Wejnarski et al. [16] confirms the results obtained, indicating significantly more frequent AEMS interventions to adult patients. However, it is important to stress the noted significant number of missions to infants and newborns, which accounted for as many as 394 cases (18.29% of all missions). The data suggest that HEMS crews are much more likely to be dispatched to the scene if the burn patient is a child under 1-year-old. In the study by Nicholson B. et al., HEMS intervention rates for burnt children were shown to be as high as 18.1% [17]. It should be noted that helicopter rescue flights are associated with risk. In the case of night time, dense development, and bad weather conditions, there is a risk for both the crew and the patient being transported. The lack of sufficient infrastructure sometimes causes the need for additional ambulance transport from the helicopter to the hospital building (no landing pad at the hospital).

Patients showed extensive body burns, as suggested by the ICD-10 code (T29—multiple body areas), which was diagnosed in 890 cases, code T21 (trunk) diagnosed in 255 cases, and T31 (extensive) in 243 cases. The AEMS physician diagnosed respiratory burns (T27) only in 3.62% of the missions (*n* = 78). Patients with such injuries usually show a more severe general condition and require the implementation of mechanical ventilation, as confirmed by a study by Cachafeiro et al. [18]. During statistical analysis, a significant relationship was found between the age group and the ICD-10 diagnosis (*p* < 0.001). The study showed that pediatric patients were significantly more likely to experience thermal and chemical burns of the torso than adult patients (5.11%). Adults, on the other hand, were significantly more likely to suffer respiratory burns than children. Having considered the foregoing, it can be concluded that inhalation burns are the domain of adult patients.

As already mentioned, maintaining treatment continuity by providing transportation to a higher reference center has an important role in rescue missions for burn patients. Some hospitals have the status of Burn Treatment Center in Poland. These dedicated treatment facilities were used in 63.74% of the cases analyzed. There are literature sources confirming the important role of air ambulance teams in allocating patients to dedicated medical facilities [19,20,21,22]. Our results confirmed this strategy to be effective, yet it is worth considering the reasons behind not transferring a patient to the Burn Treatment Center. The factors influencing this relationship are based on a number of variables, ranging from the physician’s decision at the scene to the hospital’s ability to accommodate a helicopter, which requires having an active helipad in the facility’s infrastructure. Roman J. et al. pointed out another problem concerning the economics of dispatching HEMS teams for minor burns [23]. The cost of a rescue helicopter intervention is significantly higher compared to a ground ambulance; therefore, the necessity of dispatching should always be considered.

Further results demonstrated the forms of patient transfer from the helicopter to ground medical rescue teams, which accounted for 315 cases. It can be inferred that this solution was used precisely because of the inability to land directly at the Burn Treatment Center.

The study conducted also included the burn patient treatment from the perspective of the Polish Medical Air Rescue. It was proven that effective analgesic treatment was implemented, achieving a statistically significant reduction in pain levels (*p* < 0.001). This confirms that adequate analgesia forms the basis of medical care for trauma patients [24,25,26,27]. The authors also presented the use of other pharmacological agents, including achieving sedation, relaxation, or administering pressure amines. It is reasonable to believe that the most commonly used medications (ketamine, rocuronium bromide, propofol) were used by the HEMS crew to perform RSI (rapid sequence intubation).

The AEMS physician is also qualified and authorized to perform advanced emergency procedures [28,29]. Sedation, endotracheal intubation, and mechanical ventilation were among the most commonly performed specialist procedures. The database obtained by the authors did not allow determining from which type of ground team (with or without a physician) the HEMS crew took over the patient. One may conclude that the incidents to which the rescue helicopter was called by the emergency medical team took over the patient from both teams with a physician and those only with paramedics; the latter are not yet certified to perform RSI [30]. The authors recommend further research to assess this correlation, as the number of intubations performed by HEMS crews (*n* = 289; 13.42%) appears to be significant, given that the vast majority of flights are supposed to support ground teams. Hall K. et al., in a retrospective review of burn patient records from a large Australian Helicopter Ambulance Service, found that intubation was performed in 60 of 490 cases, which is 12.24% [31]. The result is comparable to that obtained by the authors of the present study. In cases of sudden cardiac arrest, both Polish medical doctors and paramedics perform endotracheal intubation, greatly improving airway security in trauma patients [32,33]. Further training and development of local authorizations for medical personnel appear to be necessary, in order to provide professional medical assistance regardless of emergency medical team type [34,35].

Limitations of the study

The data analyzed partially covered the COVID-19 pandemic period. The authors recognize the possibility that the state of the epidemic might have affected the actual number and the true nature of incidents involving trauma patients, including those suffering burns, which required AEMS intervention [36,37]. The medical records had some information missing (e.g., determining the patient’s age), resulting in limited data in 72 cases in the patient profile section. The database also did not provide detailed information on the purpose of the patient’s transfer to the ground-based EMS team or the police. This information could be supplemented by conclusions covering the organizational and transportation activities of HEMS crews. The authors limited the analysis to medications and procedures performed exclusively by a medical doctor. Standard procedures (e.g., fluid therapy, application of topical dressings) that can also be performed by paramedics were omitted.

## 5. Conclusions

HEMS crews are more likely to have to intervene to men suffering burns than women. The predominant age group is adults; yet there is a special role for helicopter crews demonstrated for children under 1-year-old. Both pediatric and adult patients were most often diagnosed with extensive body burns, which raises the need to implement additional medical measures. In 63.74% of the missions, patients were transported to the Burn Treatment Center. The cooperation of the HEMS with ground emergency medical teams during transportation to the hospital seems to be crucial for safe, prompt and effective transfer. HEMS crews implement effective pharmacological analgesia, and handle rescue medications and procedures to stabilize the patient’s condition. The authors suggest that the results obtained should form the basis for analyzing the possibility of expanding higher-referral facilities with helipads for HEMS crews, which could significantly speed up the transfer of patients without the need for an additional transfer by ambulance.

## Figures and Tables

**Table 1:** Characteristics of burn patients during HEMS missions.

Variables	M ± SD	Mean	Range	Mo (n, %)
Age	35.05 ± 26.88	36.0	0.17–96	1 (394, 18.29%)
			N	**%**
Age group	pediatric patient		673	31.24
	adult patient		1409	65.41
	no data		72	3.34
Sex	female		570	26.46
	male		1574	73.07
	NN (no name)		10	0.46

**Table 2:** ICD-10 diagnoses among burn patients receiving AEMS intervention by age group.

ICD-10 Diagnosis (Thermal and Chemical Burns)	Age Group		Pearson’s Chi-Square Test	
	Pediatric Patient	Adult Patient	Chi^2^	*p*
T20—head and neck	89 (13.22%)	212 (15.05%)	231.736	<0.001
T21—trunk	183 (27.19%)	72 (5.11%)		
T22—shoulder and upper extremity	14 (2.08%)	20 (1.42%)		
T23—wrist and hand	11 (1.63%)	20 (1.42%)		
T24—hip and lower extremity	22 (3.27%)	43 (3.05%)		
T27—respiratory tract	6 (0.89%)	72 (5.11%)		
T29—numerous areas of the body	226 (33.58%)	664 (47.13%)		
T30—unspecified area	37 (5.50%)	96 (6.81%)		
T31—extensive	64 (9.51%)	179 (12.70%)		
Other ICD diagnosis	21 (3.12%)	31 (2.20%)		
Total patients *	673	1409		

* 72 cases did not have a specific age on file.

**Table 3:** Handing over patients during HEMS missions.

Further Management of AEMS Patients	Number [N]	Percentage [%]
Transferred to hospital	1679	77.95
Handed over to ground EMS (without a physician)	130	6.04
Handed over to ground EMS (with a physician)	185	8.59
Handed over to the Polish Medical Air Rescue airplane	36	1.67
Handed over to another AEMS team	53	2.46
Left in place	48	2.23
Handed over to the police	3	0.14
Other	20	0.93

**Table 4:** HEMS missions by target hospital in terms of age group.

Age Group	Target Hospital			Pearson’s Chi^2^ Test *
	Burn Treatment Center	Other Medical Entity	NN	
Pediatric patient	223 (33.14%)	404 (60.03%)	46 (6.84%)	Chi^2^ = 423.428; *p* < 0.001
Adult patient	1097 (77.86%)	236 (16.75%)	76 (5.39%)	
NN	53 (73.61%)	11 (15.28%)	8 (11.11%)	
Total	1373 (63.74%)	651 (30.22%)	130 (6.04%)	

* only patients for whom complete data were collected regarding age and target hospital were analyzed.

**Table 5:** Medications administered to burn patients during HEMS missions.

Medications Administered by the AEMS Crew	Number [N]	Percentage [%]
Ketamine	511	23.72
Rocuronium bromide	340	15.78
Propofol	302	14.02
Ondansetron	111	5.15
Levonor	57	2.65
Chlorsuccillin	54	2.51
Etomidate	43	2.0
Ephedrine 25 mg	23	1.07
Noradrenaline 4 mg	21	0.97
Noradrenaline 1 mg	9	0.42
Dopamine 200 mg	8	0.37
Dobutamine 250 mg	4	0.19
Tranexamic acid	3	0.14
Theophylline 300 mg	1	0.05
Tiopenthal	1	0.05

**Table 6:** Emergency procedures performed on burn patients during HEMS missions.

Procedures Performed by HEMS Crews	Number [N]	Percentage [%]
Sedation	665	30.87
Intubation	289	13.42
Mechanical ventilation	285	13.23
Relaxation	181	8.40
Urinary catheter	143	6.64
Ultrasound	53	2.46
Gastric tube	35	1.62
Chest drainage (right side)	5	0.23
Chest drainage (left side)	3	0.14

**Table 7:** The use of intubation in patients during HEMS missions.

Variable		Intubation n (%)	Pearson’s Chi^2^ Test
Age	pediatric patient	17 (2.71%)	Chi^2^ = 88.785; *p* < 0.001
	adult patient	240 (18.15%)	
Gender	female	59 (10.99%)	Chi^2^ = 6.617; *p* < 0.013
	male	225 (15.34%)	
ECG	sinus rhythm	232 (13.27%)	Chi^2^ = 13.442; *p* < 0.001
	abnormal	54 (22.04%)	
dyspnea	yes	82 (69.49%)	Chi^2^ = 307.979; *p* < 0.001
	no	191 (11.0%)	
	apnea	15 (10.64%)	
cyanosis	yes	11 (39.29%)	*p* = 0.001 *
	no	277 (14.08%)	
Skin color	normal	126 (9.89%)	Chi^2^ = 71.762; *p* < 0.001
	pale	66 (29.73%)	
	flushing	94 (18.91%)	
Variable	Intubation	Level	Welch test
RTS (Revised Trauma Score)	yes	10.76 ± 2.34	t = 4.075; *p* < 0.001
	no	11.44 ± 2.05	
GCS (Glasgow Coma Scale)	yes	12.0 ± 3.97	t = 7.855; *p* < 0.001
	no	14.16 ± 2.70	

Legend: t—Welch’s test; Chi^2^—Pearson’s Chi-square test; * due to expected frequencies less than 5, Fisher’s exact test was used.

## Data Availability

The datasets generated and analyzed during the current study are available from the corresponding author on reasonable request.
